# Anatomic variation of prescription points and treatment volume with fractionated high‐dose rate gynecological brachytherapy

**DOI:** 10.1120/jacmp.v3i1.2586

**Published:** 2002-01-01

**Authors:** Osman A. Elhanafy, Rupak K. Das, Bhudatt R. Paliwal, Mostafa D. Migahed, Hanim A. Sakr, Mostafa Elleithy

**Affiliations:** ^1^ Department of Human Oncology University of Wisconsin Madison Wisconsin; ^2^ Mansoura University Egypt

**Keywords:** brachytherapy, intracavitary, high‐dose rate, cervical carcinoma, fractionation

## Abstract

The purpose of this report is to evaluate the geometric movement (relative to the bony pelvis) and dose variation of brachytherapy reference points in the same patient at repeated high‐dose rate (HDR) intracavitary implants. A study was also concluded to find the variation in treatment volume from repeated fractions. Twenty‐five consecutive cervical cancer patients (all stages) treated with external beam and fractionated HDR intracavitary implants at the University of Wisconsin were reviewed. Each brachytherapy insertion had a different plan generated prior to treatment delivery. ICRU #38 prescription points (A, B, P, bladder, and rectum) were used. Dose volume histogram was generated and treated volume to the prescription dose was recorded for each fraction. Motion analysis of the various points (from a common origin) in subsequent fractions relative to the first fraction revealed a shift of 2–9 mm in a single plane. Vector analysis revealed the magnitude of the average shift ranged from 10–13 mm. These shifts resulted in a dose difference of >20% for the bladder and rectum points, but < than 8% for the other points. Dose volume histograms revealed that with the change in the anatomy of the cervix and upper vagina during a patient's course of treatment, the treatment volume changes considerably. Thirty‐six percent of all patients (9/16) had a reduction in the size of the ovoid during the treatment course. Sixty percent of all patients (15/25) had volume changes <10%. Sixty‐two and one half percent of patients (10/16) who did not undergo a reduction of avoid size during the entire course of the treatment had volume change <10%. Since there is a change in the anatomy of the cervix and upper vagina during the course of a treatment along with the irre‐producibility of the packing, there is movement of the absolute position of the prescription points between fractions, thus emphasizing the importance of individual dosimetry. Moreover, due to the same reasons, there are significant changes in the treatment volume among implants for the same patient. Volume reduction caused by reduction in ovoid size alone could not be extracted from this study.

PACS number(s): 87.53.–j

## INTRODUCTION

The combination of external beam radiation and intracavitary brachytherapy has been well established in the definitive management of cervical cancer. High‐dose rate (HDR) brachytherapy appears to be comparable to low‐dose rate (LDR) brachytherapy. The advantages and disadvantages of HDR to LDR have been extensively reviewed.^1^ ^–^ ^4^ Reflecting the HDR radiobiological effects, number of fractions between institutions range from 2 to 16^2^ Petereit and Pearcy recently published a fractionation analysis and concluded that five HDR fractions with a dose of 5–6 Gy appeared safe and efficacious.^5^ Since the anatomy changes from one fraction to the next, planning and dose optimization has been recommended for every fraction.^6^ ^–^ ^8^ Hoskin [6] *et al.* found major variations within the same patient in craniocaudal and antero‐posterior positioning of the applicators. They reported that the anterior rectal wall moved an average of 10.5 mm (range 0‐22 mm) in relation to the ovoid surface between the first and second fractions. Kim *et al.* ^9^ evaluated geometric variations in multiple intracavitary applications. They reported that major variations (defined as more than 1‐cm deviation) occurred more commonly in the colpostats than in the tandem, attributing to the fact that the variation was caused by vaginal packing. A literature review indicates that no extensive study has quantified dose differences to critical structures for each fraction in high‐dose rate intracavitary implants. Hoskin *et al* ^6^ calculated changes in dose to the anterior rectal wall between two insertions. They reported that since this is a region of high‐dose gradient, a median of 10.5‐mm shift of the rectal wall has major implications for dosimetry. Moreover, since the rectal wall is probably the major limiting normal tissue in gynecological brachytherapy, they suggested that individual dosimetry for each high‐dose rate insertion is required to define the dose both to the tumor area and more critically to limiting normal tissue structures within the treatment area.

The purpose of this report is to evaluate the geometric movement of fixed reference points in patients receiving fractionated gynecological HDR brachytherapy, and to determine the actual dose difference to reference points and critical structures for each insertion. Since anatomical changes and differences in packing results among various fractions, the volume enclosed by a specified isodose surface arising from an intracavitary implant was determined, since it may correlate with clinical outcome.^10^ Treatment volume encompassed by the prescription isodose surface was also calculated for this study. Variation in treatment volume between fractions was also calculated to determine correlation with disease stage.

## MATERIAL AND METHODS

We reviewed radiation therapy treatment planning results of 25 consecutive patients of all stages of cervical carcinoma treated from November 1998 to August 1999 at the University of Wisconsin (Table I). Concurrent with HDR brachytherapy, all patients received external beam radiation (four‐field box, 45 Gy total). HDR dose fractionation scheme (Table I) for intracavitary implants was dependent on patient age, lesion size, disease stage (determines pathologically), and cumulative dose to the bladder and rectum.^7^ A total of 119 insertions were analyzed. The standard Nucletron^a^ applicator set without shielding was used, and the 30° tandem with small ovoids (2‐cm diameter) was most commonly employed. The following ICRU #38^11^ prescription points were used: point A, B, P, bladder, and rectum.

**Table I:** Patient characteristics and fractionation scheme.

Patient characteristic		Fractionation scheme	
Stage	No. of patients	No. of patients	No. of fractions
Ib	10	2	3
IIb	14	2	4
IIIb	1	21	5

The procedural and physical considerations of the Madison system of HDR intracavitary brachytherapy for carcinoma of the cervix has been detailed elsewhere.^8^ Nucletron treatment planning system (NPS version 11.43) was used for this study. Dose points as explained in Ref. [8] were used for polynomial optimization on distance. The smallest dwell time gradient restriction that did not result in negative dwell time was used for dose optimization.^12^

For each fraction, standard antero‐posterior and lateral radiographs were taken with fixed magnification factors. The following ICRU #38 prescription points were used: bladder, rectum, and points A, B, and P.^11^ Point A is 2‐cm lateral to the midline of the intrauterine canal and 2‐cm cephalad to the external cervical os.^13^ ^,^ ^14^ Points B and P lie 5 and 6 cm, respectively, to the right and left of the patient's midline in the transverse plane. Bladder and rectal points are in accordance with #38.^11^ Using the bony landmark in the pelvis as defined in each fraction, comparison of the spatial position of the reference points was performed. In the coronal and the sagittal plane, the origin was defined as the midpoint of the line joining the superior portion of the right and left acetabulum. The coordinates of the prescription points for each fraction were determined relative to this origin. Spatial movement of the prescription points from the first to the subsequent implants was then determined in each plane (*x, y*, and *z*) by vector analysis and correlated to the dose difference.

A post treatment, three‐dimensional dose volume study was performed for all patients. Since treatment plans were calculated with the dose distribution origin at 2‐cm cephalad to the external cervical os and on the applicator (tandem), a rectangular volume of $10\times 8\times 6{\;\text{cm}}^{3}$ was sufficient to cover the whole prescription isodose surface. One hundred thousand points were used to calculate the dose volume histogram. The treatment volume encompassed by the 100% prescription isodose surface was then recorded. The average and standard deviation isodose volumes for each patient was calculated for repeated insertions.

## RESULTS

The frequency distribution of ovoid size employed in each fraction is tabulated in Table II. For each fraction the largest possible ovoid size that fits in the cavity was used. Two patients had a reduction of size from medium (2.5‐cm diameter) to small (2‐cm diameter) and seven had a reduction of small to minimum (1.6‐cm diameter). The remaining 16 did not have any ovoid size reduction during the course of their treatment. In no case were large ovoids (3‐cm diameter) used.

**Table II:** Frequency distribution of ovoid size with implant number.

	Implant number				
Ovoid size	1	2	3	4	5
Medium	5	3	3	2	2
Small	18	17	16	15	14
Mini	2	5	6	6	5

The average displacement of the prescription points relative to the bony pelvis in the 3 orthogonal axis from the first to the subsequent insertions is shown in Table III. The displacement of the individual points ranged from 2 to 9 mm along a given plane. The mean of the total displacement is also tabulated in Table III. For the rectum, bladder, and points B and P, the average displacement is ~13mm. Since in the current treatment planning system, point A coordinates are specified as applicator points with respect to the external os, the individual displacement of these two points are equal. Our findings of the movement of the prescription points agree with those of Grigsby *et al*, who did a similar study for LDR intracavitary implants.^15^ They reported an average shift of 10 to 13 mm of the individual points from the second implant relative to the first implant.

**Table III:** Average displacement of prescription points for 25 patients receiving 119 fractions.

	Average displacement (mm)			
Prescription points	ΔX	ΔY	ΔZ	$\bar{\Delta R}={\left(\Delta X^{2}+\Delta Y^{2}+\Delta Z^{2}\right)}^{0.5}$
Bladder	5.8	5.0	7.4	12.2
Rectum	5.4	8.0	8.3	14.6
Rt. A	2.7	5.2	3.8	7.9
Lt. A	2.7	5.2	3.8	7.9
Rt. B	2.7	8.9	7.4	13.2
Lt. B	2.5	8.2	8.2	13.4
Rt. P	3.0	9.1	7.6	13.6
Lt. P	2.6	8.4	8.6	13.8

Table IV demonstrates the effect of movement on dose to the prescription points. No attempt was made to account for the reduction in the ovoid size that might have occurred during the course of the treatment. The average change in the bladder and rectum dose was approximately 15% and 13%, respectively, of the prescription dose (typical prescription dose is 6 Gy). The average variation in dose to points B and P was between 2% to 5% with a maximum deviation of 8%, but the maximum dose deviation of the bladder and rectum was as high as 25% (which is 1.5 Gy for a prescription dose of 6 Gy). Since the 100% prescription dose was prescribed to point A and planning for every fraction was done to optimize the dose point, point A was not included in Table IV.

**Table IV:** Average dose difference for prescription points as a percentage of prescription dose.

	Bladder	Rectum	Rt. B	Lt. B	Rt. P	Lt. P
Average	14.6±11.2	12.9±9.4	4.4±3.6	3.6±2.7	3.2±2.5	2.6±2.1
Maximum	25.8	22.3	8.0	6.3	5.7	4.7
Minimum	3.4	3.5	0.87	0.83	0.74	0.48

Table V details changes to the treatment volume resulting from repeated fractions. As can be seen from Table V, with the change in the anatomy of the cervix and upper vagina during a patient's course of treatment, the treatment volume changes considerably. Sixty percent (15/25) of the patients had a volume change of less than 10% of its average value, while the rest 40% of the patient had a change of volume greater than 10% of its average value. Sixty‐two and one half percent (10/16) of patients who did not undergo a reduction of the ovoid size during the course of the treatment had a volume change of less than 10%. The reduction in the volume resulted either from the ovoid size reduction or the separation of the ovoids. Table V also documents the change in treatment volume with disease stage, along with the reduction of ovoid size during the course of a treatment. Sixty percent of the patients, where the volume change was less than 10%, belonged to Stage Ib and IIb. As can be seen, one Stage IIb patient without a reduction in the ovoid size had the largest volume change greater than 15%, while a Stage IIIb patient with ovoid size (from medium to small) had a volume change greater than 20%.

**Table V:** Frequency distribution of change in treatment volume with staging and ovoid size reduction.

Change in volume (%)	Total no. of patients	Ovoid size reduction	Stage
0–5	5	no reduction	4×Ib and 1×IIb
	1	reduction	1×Ib
5–10	5	no reduction	3×Ib and 2×IIb
	4	reduction	3×IIb and 1×Ib
10–15	5	no reduction	4×IIb and 1×IIb
	3	reduction	3×IIb
15–20	1	no reduction	IIb
>20	1	reduction	IIIb

## CONCLUSION

The current analysis demonstrates significant applicator motion upon repeated fractions in HDR fractionated intracavitary implants. Moreover, anatomical changes in the upper vagina alter the vaginal packing, which in turn perturb dose to the prescription points. Based upon these observations, it is warranted that optimized treatment planning be performed for every fraction. Moreover, the same reason that attributes to the change in the standard prescription points from repeated fractions gives rise to a major change in the treatment volume. For higher stage patients (IIb, IIIb), there is a greater probability that there will be some anatomical change in the upper vagina, giving rise to a significant change in the treatment volume. Volume reduction caused by reduction in ovoid size alone could not be extracted from this study.
